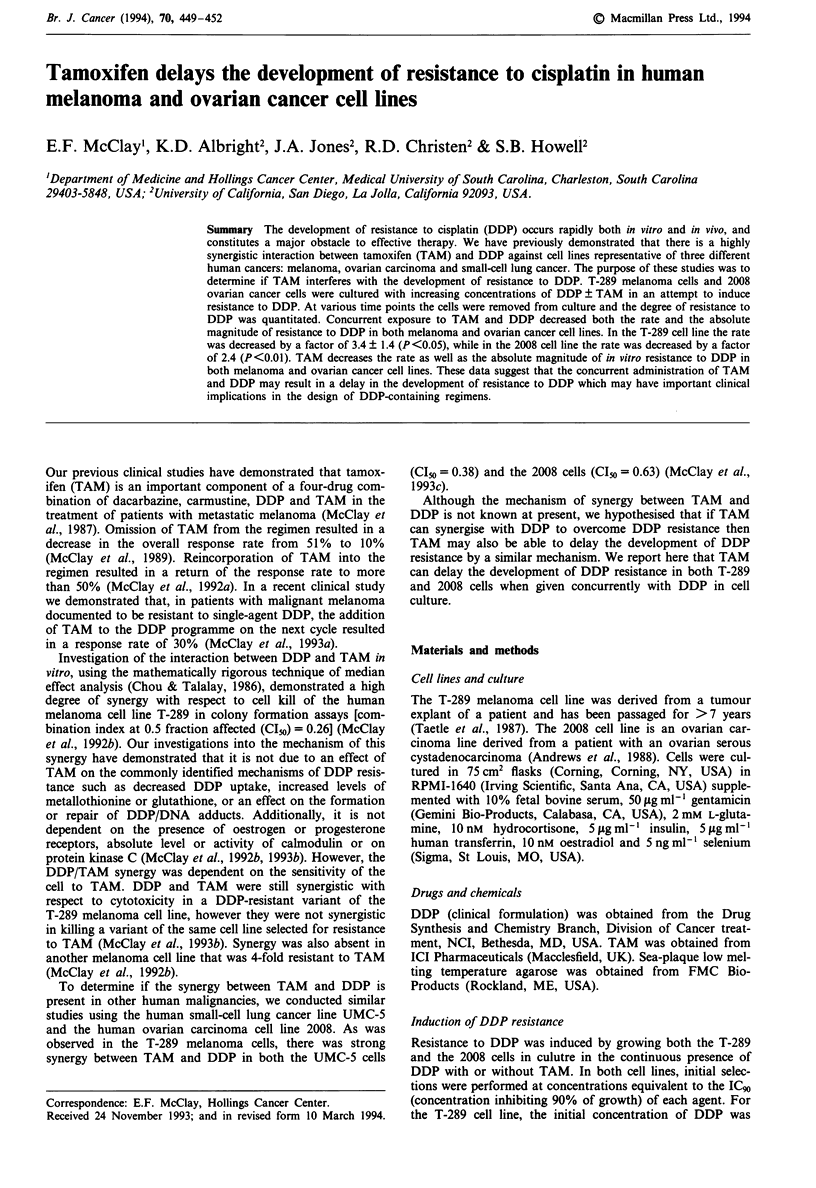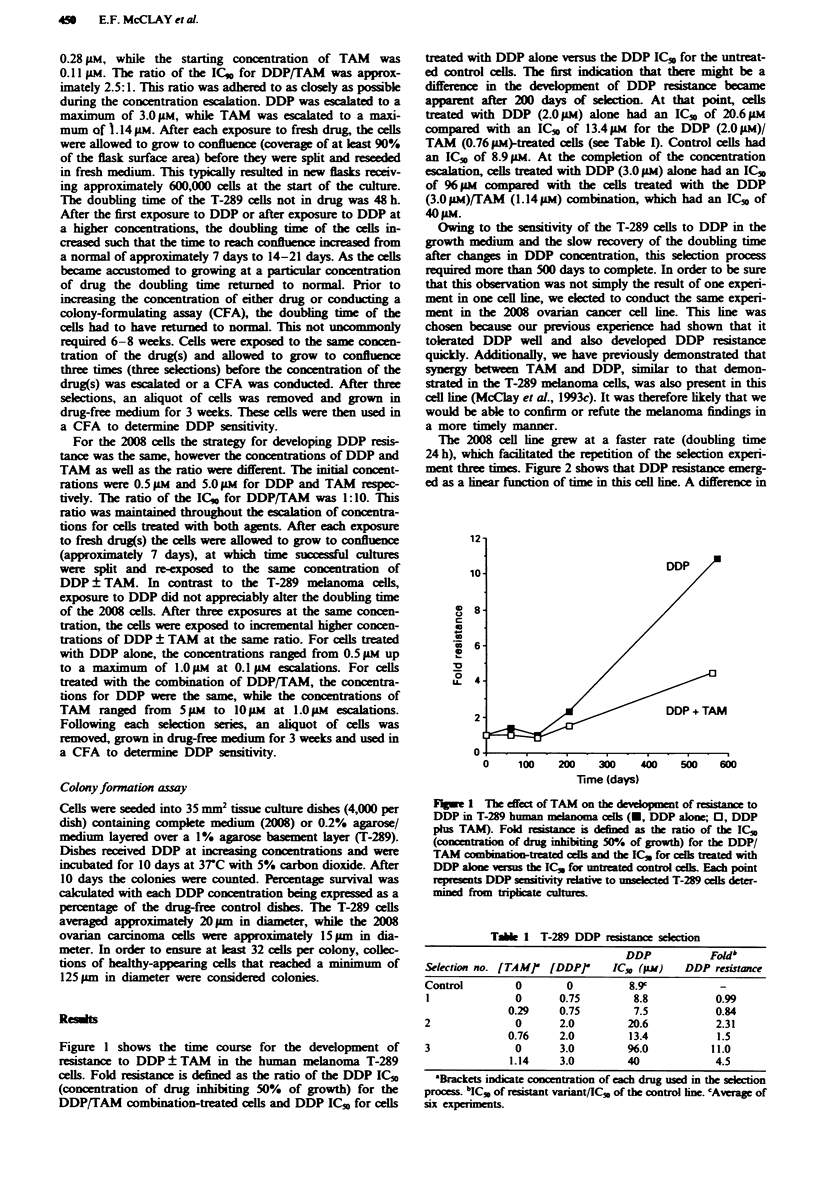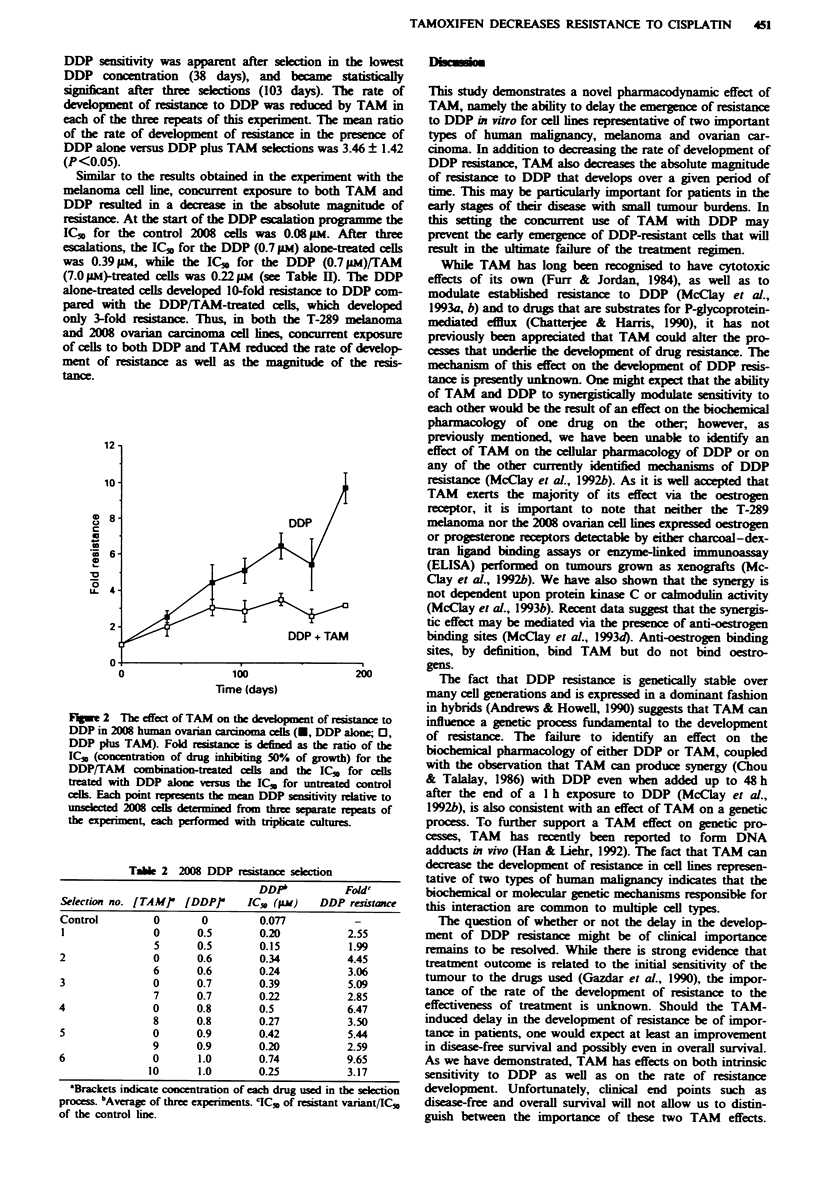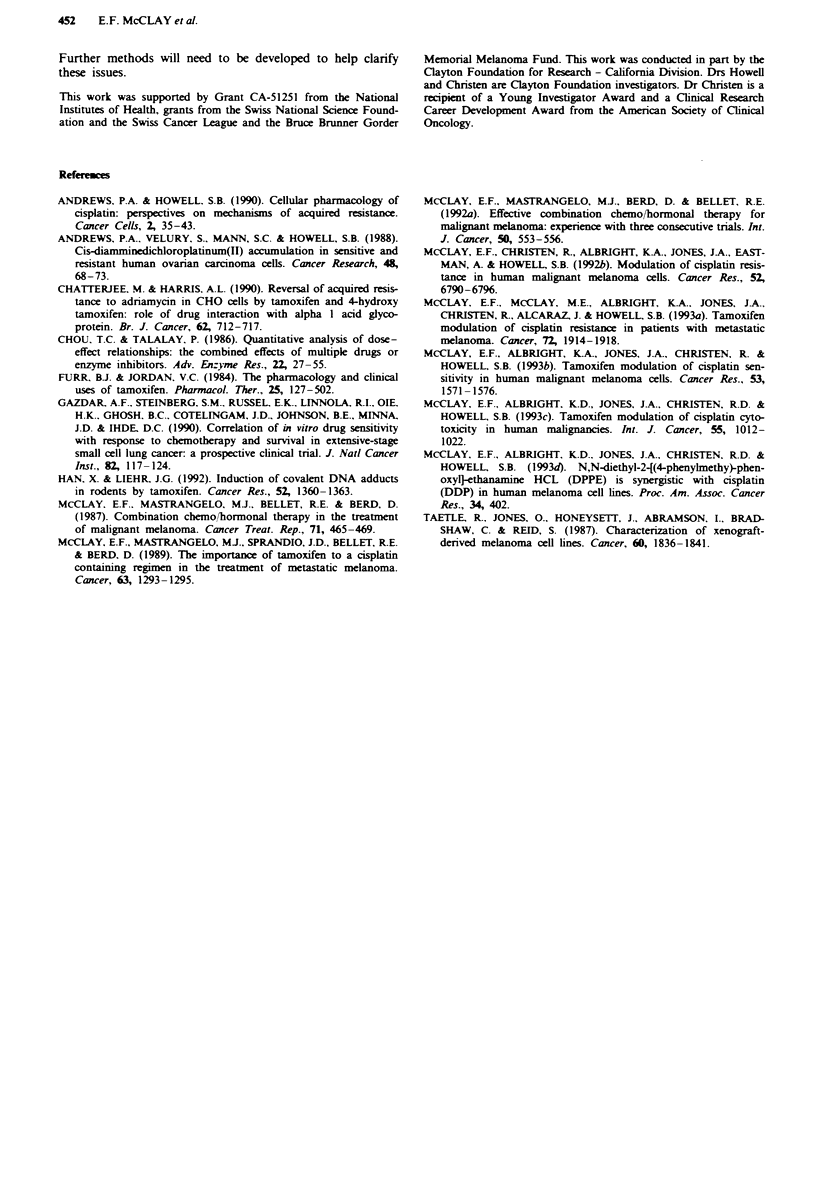# Tamoxifen delays the development of resistance to cisplatin in human melanoma and ovarian cancer cell lines.

**DOI:** 10.1038/bjc.1994.326

**Published:** 1994-09

**Authors:** E. F. McClay, K. D. Albright, J. A. Jones, R. D. Christen, S. B. Howell

**Affiliations:** Department of Medicine, Medical University of South Carolina, Charleston 29403-5848.

## Abstract

The development of resistance to cisplatin (DDP) occurs rapidly both in vitro and in vivo, and constitutes a major obstacle to effective therapy. We have previously demonstrated that there is a highly synergistic interaction between tamoxifen (TAM) and DDP against cell lines representative of three different human cancers: melanoma, ovarian carcinoma and small-cell lung cancer. The purpose of these studies was to determine if TAM interferes with the development of resistance to DDP. T-289 melanoma cells and 2008 ovarian cancer cells were cultured with increasing concentrations of DDP +/- TAM in an attempt to induce resistance to DDP. At various time points the cells were removed from culture and the degree of resistance to DDP was quantitated. Concurrent exposure to TAM and DDP decreased both the rate and the absolute magnitude of resistance to DDP in both melanoma and ovarian cancer cell lines. In the T-289 cell line the rate was decreased by a factor of 3.4 +/- 1.4 (P < 0.05), while in the 2008 cell line the rate was decreased by a factor of 2.4 (P < 0.01). TAM decreases the rate as well as the absolute magnitude of in vitro resistance to DDP in both melanoma and ovarian cancer cell lines. These data suggest that the concurrent administration of TAM and DDP may result in a delay in the development of resistance to DDP which may have important clinical implications in the design of DDP-containing regimens.


Br. J. Cancer (1994), 70, 449-452 © Macmillan Press Ltd., 1994

### Tamoxifen delays the development of resistance to cisplatin in human melanoma and ovarian cancer cell lines

E.F. McClay[1], K.D. Albright[2], J.A. Jones[2], R.D. Christen[2] & S.B. Howell[2]

[1]Department of Medicine and Hollings Cancer Center, Medical University of South Carolina, Charleston, South Carolina 29403-5848, USA; [2]University of California, San Diego, La Jolla, California 92093, USA.

**Summary** The development of resistance to cisplatin (DDP) occurs rapidly both *in vitro* and *in vivo*, and constitutes a major obstacle to effective therapy. We have previously demonstrated that there is a highly synergistic interaction between tamoxifen (TAM) and DDP against cell lines representative of three different human cancers: melanoma, ovarian carcinoma and small-cell lung cancer. The purpose of these studies was to determine if TAM interferes with the development of resistance to DDP. T-289 melanoma cells and 2008 ovarian cancer cells were cultured with increasing concentrations of DDP ± TAM in an attempt to induce resistance to DDP. At various time points the cells were removed from culture and the degree of resistance to DDP was quantitated. Concurrent exposure to TAM and DDP decreased both the rate and the absolute magnitude of resistance to DDP in both melanoma and ovarian cancer cell lines. In the T-289 cell line the rate was decreased by a factor of 3.4 ± 1.4 ($P<0.05$), while in the 2008 cell line the rate was decreased by a factor of 2.4 ($P<0.01$). TAM decreases the rate as well as the absolute magnitude of *in vitro* resistance to DDP in both melanoma and ovarian cancer cell lines. These data suggest that the concurrent administration of TAM and DDP may result in a delay in the development of resistance to DDP which may have important clinical implications in the design of DDP-containing regimens.

Our previous clinical studies have demonstrated that tamoxifen (TAM) is an important component of a four-drug combination of dacarbazine, carmustine, DDP and TAM in the treatment of patients with metastatic melanoma (McClay *et al.*, 1987). Omission of TAM from the regimen resulted in a decrease in the overall response rate from 51% to 10% (McClay *et al.*, 1989). Reincorporation of TAM into the regimen resulted in a return of the response rate to more than 50% (McClay *et al.*, 1992*a*). In a recent clinical study we demonstrated that, in patients with malignant melanoma documented to be resistant to single-agent DDP, the addition of TAM to the DDP programme on the next cycle resulted in a response rate of 30% (McClay *et al.*, 1993*a*).

Investigation of the interaction between DDP and TAM *in vitro*, using the mathematically rigorous technique of median effect analysis (Chou & Talalay, 1986), demonstrated a high degree of synergy with respect to cell kill of the human melanoma cell line T-289 in colony formation assays [combination index at 0.5 fraction affected ($CI_{50}$) = 0.26] (McClay *et al.*, 1992*b*). Our investigations into the mechanism of this synergy have demonstrated that it is not due to an effect of TAM on the commonly identified mechanisms of DDP resistance such as decreased DDP uptake, increased levels of metallothionine or glutathione, or an effect on the formation or repair of DDP/DNA adducts. Additionally, it is not dependent on the presence of oestrogen or progesterone receptors, absolute level or activity of calmodulin or on protein kinase C (McClay *et al.*, 1992*b*, 1993*b*). However, the DDP/TAM synergy was dependent on the sensitivity of the cell to TAM. DDP and TAM were still synergistic with respect to cytotoxicity in a DDP-resistant variant of the T-289 melanoma cell line, however they were not synergistic in killing a variant of the same cell line selected for resistance to TAM (McClay *et al.*, 1993*b*). Synergy was also absent in another melanoma cell line that was 4-fold resistant to TAM (McClay *et al.*, 1992*b*).

To determine if the synergy between TAM and DDP is present in other human malignancies, we conducted similar studies using the human small-cell lung cancer line UMC-5 and the human ovarian carcinoma cell line 2008. As was observed in the T-289 melanoma cells, there was strong synergy between TAM and DDP in both the UMC-5 cells ($CI_{50}$ = 0.38) and the 2008 cells ($CI_{50}$ = 0.63) (McClay *et al.*, 1993*c*).

Although the mechanism of synergy between TAM and DDP is not known at present, we hypothesised that if TAM can synergise with DDP to overcome DDP resistance then TAM may also be able to delay the development of DDP resistance by a similar mechanism. We report here that TAM can delay the development of DDP resistance in both T-289 and 2008 cells when given concurrently with DDP in cell culture.

### Materials and methods

### Cell lines and culture

The T-289 melanoma cell line was derived from a tumour explant of a patient and has been passaged for >7 years (Taetle *et al.*, 1987). The 2008 cell line is an ovarian carcinoma line derived from a patient with an ovarian serous cystadenocarcinoma (Andrews *et al.*, 1988). Cells were cultured in 75 $cm^2$ flasks (Corning, Corning, NY, USA) in RPMI-1640 (Irving Scientific, Santa Ana, CA, USA) supplemented with 10% fetal bovine serum, 50 $\mu g\ ml^{-1}$ gentamicin (Gemini Bio-Products, Calabasa, CA, USA), 2 mM L-glutamine, 10 nM hydrocortisone, 5 $\mu g\ ml^{-1}$ insulin, 5 $\mu g\ ml^{-1}$ human transferrin, 10 nM oestradiol and 5 $ng\ ml^{-1}$ selenium (Sigma, St Louis, MO, USA).

### Drugs and chemicals

DDP (clinical formulation) was obtained from the Drug Synthesis and Chemistry Branch, Division of Cancer treatment, NCI, Bethesda, MD, USA. TAM was obtained from ICI Pharmaceuticals (Macclesfield, UK). Sea-plaque low melting temperature agarose was obtained from FMC Bio-Products (Rockland, ME, USA).

### Induction of DDP resistance

Resistance to DDP was induced by growing both the T-289 and the 2008 cells in culutre in the continuous presence of DDP with or without TAM. In both cell lines, initial selections were performed at concentrations equivalent to the $IC_{90}$ (concentration inhibiting 90% of growth) of each agent. For the T-289 cell line, the initial concentration of DDP was

Correspondence: E.F. McClay, Hollings Cancer Center.
Received 24 November 1993; and in revised form 10 March 1994.

0.28 μM, while the starting concentration of TAM was 0.11 μM. The ratio of the $IC_{90}$ for DDP/TAM was approximately 2.5:1. This ratio was adhered to as closely as possible during the concentration escalation. DDP was escalated to a maximum of 3.0 μM, while TAM was escalated to a maximum of 1.14 μM. After each exposure to fresh drug, the cells were allowed to grow to confluence (coverage of at least 90% of the flask surface area) before they were split and reseeded in fresh medium. This typically resulted in new flasks receiving approximately 600,000 cells at the start of the culture. The doubling time of the T-289 cells not in drug was 48 h. After the first exposure to DDP or after exposure to DDP at a higher concentrations, the doubling time of the cells increased such that the time to reach confluence increased from a normal of approximately 7 days to 14–21 days. As the cells became accustomed to growing at a particular concentration of drug the doubling time returned to normal. Prior to increasing the concentration of either drug or conducting a colony-formulating assay (CFA), the doubling time of the cells had to have returned to normal. This not uncommonly required 6–8 weeks. Cells were exposed to the same concentration of the drug(s) and allowed to grow to confluence three times (three selections) before the concentration of the drug(s) was escalated or a CFA was conducted. After three selections, an aliquot of cells was removed and grown in drug-free medium for 3 weeks. These cells were then used in a CFA to determine DDP sensitivity.

For the 2008 cells the strategy for developing DDP resistance was the same, however the concentrations of DDP and TAM as well as the ratio were different. The initial concentrations were 0.5 μM and 5.0 μM for DDP and TAM respectively. The ratio of the $IC_{90}$ for DDP/TAM was 1:10. This ratio was maintained throughout the escalation of concentrations for cells treated with both agents. After each exposure to fresh drug(s) the cells were allowed to grow to confluence (approximately 7 days), at which time successful cultures were split and re-exposed to the same concentration of DDP ± TAM. In contrast to the T-289 melanoma cells, exposure to DDP did not appreciably alter the doubling time of the 2008 cells. After three exposures at the same concentration, the cells were exposed to incremental higher concentrations of DDP ± TAM at the same ratio. For cells treated with DDP alone, the concentrations ranged from 0.5 μM up to a maximum of 1.0 μM at 0.1 μM escalations. For cells treated with the combination of DDP/TAM, the concentrations for DDP were the same, while the concentrations of TAM ranged from 5 μM to 10 μM at 1.0 μM escalations. Following each selection series, an aliquot of cells was removed, grown in drug-free medium for 3 weeks and used in a CFA to determine DDP sensitivity.

*Colony formation assay*

Cells were seeded into 35 $mm^2$ tissue culture dishes (4,000 per dish) containing complete medium (2008) or 0.2% agarose/medium layered over a 1% agarose basement layer (T-289). Dishes received DDP at increasing concentrations and were incubated for 10 days at 37°C with 5% carbon dioxide. After 10 days the colonies were counted. Percentage survival was calculated with each DDP concentration being expressed as a percentage of the drug-free control dishes. The T-289 cells averaged approximately 20 μm in diameter, while the 2008 ovarian carcinoma cells were approximately 15 μm in diameter. In order to ensure at least 32 cells per colony, collections of healthy-appearing cells that reached a minimum of 125 μm in diameter were considered colonies.

### Results

Figure 1 shows the time course for the development of resistance to DDP ± TAM in the human melanoma T-289 cells. Fold resistance is defined as the ratio of the DDP $IC_{50}$ (concentration of drug inhibiting 50% of growth) for the DDP/TAM combination-treated cells and DDP $IC_{50}$ for cells treated with DDP alone versus the DDP $IC_{50}$ for the untreated control cells. The first indication that there might be a difference in the development of DDP resistance became apparent after 200 days of selection. At that point, cells treated with DDP (2.0 μM) alone had an $IC_{50}$ of 20.6 μM compared with an $IC_{50}$ of 13.4 μM for the DDP (2.0 μM)/TAM (0.76 μM)-treated cells (see Table I). Control cells had an $IC_{50}$ of 8.9 μM. At the completion of the concentration escalation, cells treated with DDP (3.0 μM) alone had an $IC_{50}$ of 96 μM compared with the cells treated with the DDP (3.0 μM)/TAM (1.14 μM) combination, which had an $IC_{50}$ of 40 μM.

Owing to the sensitivity of the T-289 cells to DDP in the growth medium and the slow recovery of the doubling time after changes in DDP concentration, this selection process required more than 500 days to complete. In order to be sure that this observation was not simply the result of one experiment in one cell line, we elected to conduct the same experiment in the 2008 ovarian cancer cell line. This line was chosen because our previous experience had shown that it tolerated DDP well and also developed DDP resistance quickly. Additionally, we have previously demonstrated that synergy between TAM and DDP, similar to that demonstrated in the T-289 melanoma cells, was also present in this cell line (McClay *et al.*, 1993*c*). It was therefore likely that we would be able to confirm or refute the melanoma findings in a more timely manner.

The 2008 cell line grew at a faster rate (doubling time 24 h), which facilitated the repetition of the selection experiment three times. Figure 2 shows that DDP resistance emerged as a linear function of time in this cell line. A difference in

**Figure 1** The effect of TAM on the development of resistance to DDP in T-289 human melanoma cells (■, DDP alone; □, DDP plus TAM). Fold resistance is defined as the ratio of the $IC_{50}$ (concentration of drug inhibiting 50% of growth) for the DDP/TAM combination-treated cells and the $IC_{50}$ for cells treated with DDP alone versus the $IC_{50}$ for untreated control cells. Each point represents DDP sensitivity relative to unselected T-289 cells determined from triplicate cultures.

**Table I** T-289 DDP resistance selection

| *Selection no.* | *[TAM]*[a] | *[DDP]*[a] | *DDP $IC_{50}$ (μM)* | *Fold*[b] *DDP resistance* |
|---|---|---|---|---|
| Control | 0 | 0 | 8.9[c] | – |
| 1 | 0 | 0.75 | 8.8 | 0.99 |
| | 0.29 | 0.75 | 7.5 | 0.84 |
| 2 | 0 | 2.0 | 20.6 | 2.31 |
| | 0.76 | 2.0 | 13.4 | 1.5 |
| 3 | 0 | 3.0 | 96.0 | 11.0 |
| | 1.14 | 3.0 | 40 | 4.5 |

[a]Brackets indicate concentration of each drug used in the selection process. [b]$IC_{50}$ of resistant variant/$IC_{50}$ of the control line. [c]Average of six experiments.

DDP sensitivity was apparent after selection in the lowest DDP concentration (38 days), and became statistically significant after three selections (103 days). The rate of development of resistance to DDP was reduced by TAM in each of the three repeats of this experiment. The mean ratio of the rate of development of resistance in the presence of DDP alone versus DDP plus TAM selections was 3.46 ± 1.42 ($P<0.05$).

Similar to the results obtained in the experiment with the melanoma cell line, concurrent exposure to both TAM and DDP resulted in a decrease in the absolute magnitude of resistance. At the start of the DDP escalation programme the $IC_{50}$ for the control 2008 cells was 0.08 µM. After three escalations, the $IC_{50}$ for the DDP (0.7 µM) alone-treated cells was 0.39 µM, while the $IC_{50}$ for the DDP (0.7 µM)/TAM (7.0 µM)-treated cells was 0.22 µM (see Table II). The DDP alone-treated cells developed 10-fold resistance to DDP compared with the DDP/TAM-treated cells, which developed only 3-fold resistance. Thus, in both the T-289 melanoma and 2008 ovarian carcinoma cell lines, concurrent exposure of cells to both DDP and TAM reduced the rate of development of resistance as well as the magnitude of the resistance.

**Figure 2** The effect of TAM on the development of resistance to DDP in 2008 human ovarian carcinoma cells (■, DDP alone; □, DDP plus TAM). Fold resistance is defined as the ratio of the $IC_{50}$ (concentration of drug inhibiting 50% of growth) for the DDP/TAM combination-treated cells and the $IC_{50}$ for cells treated with DDP alone versus the $IC_{50}$ for untreated control cells. Each point represents the mean DDP sensitivity relative to unselected 2008 cells determined from three separate repeats of the experiment, each performed with triplicate cultures.

**Table 2** 2008 DDP resistance selection

| *Selection no.* | *[TAM]*[a] | *[DDP]*[a] | *DDP*[b] *$IC_{50}$ (µM)* | *Fold*[c] *DDP resistance* |
|---|---|---|---|---|
| Control | 0 | 0 | 0.077 | – |
| 1 | 0 | 0.5 | 0.20 | 2.55 |
| | 5 | 0.5 | 0.15 | 1.99 |
| 2 | 0 | 0.6 | 0.34 | 4.45 |
| | 6 | 0.6 | 0.24 | 3.06 |
| 3 | 0 | 0.7 | 0.39 | 5.09 |
| | 7 | 0.7 | 0.22 | 2.85 |
| 4 | 0 | 0.8 | 0.5 | 6.47 |
| | 8 | 0.8 | 0.27 | 3.50 |
| 5 | 0 | 0.9 | 0.42 | 5.44 |
| | 9 | 0.9 | 0.20 | 2.59 |
| 6 | 0 | 1.0 | 0.74 | 9.65 |
| | 10 | 1.0 | 0.25 | 3.17 |

[a]Brackets indicate concentration of each drug used in the selection process. [b]Average of three experiments. [c]$IC_{50}$ of resistant variant/$IC_{50}$ of the control line.

### Discussion

This study demonstrates a novel pharmacodynamic effect of TAM, namely the ability to delay the emergence of resistance to DDP *in vitro* for cell lines representative of two important types of human malignancy, melanoma and ovarian carcinoma. In addition to decreasing the rate of development of DDP resistance, TAM also decreases the absolute magnitude of resistance to DDP that develops over a given period of time. This may be particularly important for patients in the early stages of their disease with small tumour burdens. In this setting the concurrent use of TAM with DDP may prevent the early emergence of DDP-resistant cells that will result in the ultimate failure of the treatment regimen.

While TAM has long been recognised to have cytotoxic effects of its own (Furr & Jordan, 1984), as well as to modulate established resistance to DDP (McClay *et al.*, 1993*a*, *b*) and to drugs that are substrates for P-glycoprotein-mediated efflux (Chatterjee & Harris, 1990), it has not previously been appreciated that TAM could alter the processes that underlie the development of drug resistance. The mechanism of this effect on the development of DDP resistance is presently unknown. One might expect that the ability of TAM and DDP to synergistically modulate sensitivity to each other would be the result of an effect on the biochemical pharmacology of one drug on the other; however, as previously mentioned, we have been unable to identify an effect of TAM on the cellular pharmacology of DDP or on any of the other currently identified mechanisms of DDP resistance (McClay *et al.*, 1992*b*). As it is well accepted that TAM exerts the majority of its effect via the oestrogen receptor, it is important to note that neither the T-289 melanoma nor the 2008 ovarian cell lines expressed oestrogen or progesterone receptors detectable by either charcoal–dextran ligand binding assays or enzyme-linked immunoassay (ELISA) performed on tumours grown as xenografts (McClay *et al.*, 1992*b*). We have also shown that the synergy is not dependent upon protein kinase C or calmodulin activity (McClay *et al.*, 1993*b*). Recent data suggest that the synergistic effect may be mediated via the presence of anti-oestrogen binding sites (McClay *et al.*, 1993*d*). Anti-oestrogen binding sites, by definition, bind TAM but do not bind oestrogens.

The fact that DDP resistance is genetically stable over many cell generations and is expressed in a dominant fashion in hybrids (Andrews & Howell, 1990) suggests that TAM can influence a genetic process fundamental to the development of resistance. The failure to identify an effect on the biochemical pharmacology of either DDP or TAM, coupled with the observation that TAM can produce synergy (Chou & Talalay, 1986) with DDP even when added up to 48 h after the end of a 1 h exposure to DDP (McClay *et al.*, 1992*b*), is also consistent with an effect of TAM on a genetic process. To further support a TAM effect on genetic processes, TAM has recently been reported to form DNA adducts *in vivo* (Han & Liehr, 1992). The fact that TAM can decrease the development of resistance in cell lines representative of two types of human malignancy indicates that the biochemical or molecular genetic mechanisms responsible for this interaction are common to multiple cell types.

The question of whether or not the delay in the development of DDP resistance might be of clinical importance remains to be resolved. While there is strong evidence that treatment outcome is related to the initial sensitivity of the tumour to the drugs used (Gazdar *et al.*, 1990), the importance of the rate of the development of resistance to the effectiveness of treatment is unknown. Should the TAM-induced delay in the development of resistance be of importance in patients, one would expect at least an improvement in disease-free survival and possibly even in overall survival. As we have demonstrated, TAM has effects on both intrinsic sensitivity to DDP as well as on the rate of resistance development. Unfortunately, clinical end points such as disease-free and overall survival will not allow us to distinguish between the importance of these two TAM effects.

Further methods will need to be developed to help clarify these issues.

This work was supported by Grant CA-51251 from the National Institutes of Health, grants from the Swiss National Science Foundation and the Swiss Cancer League and the Bruce Brunner Gorder Memorial Melanoma Fund. This work was conducted in part by the Clayton Foundation for Research - California Division. Drs Howell and Christen are Clayton Foundation investigators. Dr Christen is a recipient of a Young Investigator Award and a Clinical Research Career Development Award from the American Society of Clinical Oncology.

### References

ANDREWS, P.A. & HOWELL, S.B. (1990). Cellular pharmacology of cisplatin: perspectives on mechanisms of acquired resistance. *Cancer Cells*, **2**, 35-43.

ANDREWS, P.A., VELURY, S., MANN, S.C. & HOWELL, S.B. (1988). Cis-diamminedichloroplatinum(II) accumulation in sensitive and resistant human ovarian carcinoma cells. *Cancer Research*, **48**, 68-73.

CHATTERJEE, M. & HARRIS, A.L. (1990). Reversal of acquired resistance to adriamycin in CHO cells by tamoxifen and 4-hydroxy tamoxifen: role of drug interaction with alpha 1 acid glycoprotein. *Br. J. Cancer*, **62**, 712-717.

CHOU, T.C. & TALALAY, P. (1986). Quantitative analysis of dose-effect relationships: the combined effects of multiple drugs or enzyme inhibitors. *Adv. Enzyme Res.*, **22**, 27-55.

FURR, B.J. & JORDAN, V.C. (1984). The pharmacology and clinical uses of tamoxifen. *Pharmacol. Ther.*, **25**, 127-502.

GAZDAR, A.F., STEINBERG, S.M., RUSSEL, E.K., LINNOLA, R.I., OIE, H.K., GHOSH, B.C., COTELINGAM, J.D., JOHNSON, B.E., MINNA, J.D. & IHDE, D.C. (1990). Correlation of *in vitro* drug sensitivity with response to chemotherapy and survival in extensive-stage small cell lung cancer: a prospective clinical trial. *J. Natl Cancer Inst.*, **82**, 117-124.

HAN, X. & LIEHR, J.G. (1992). Induction of covalent DNA adducts in rodents by tamoxifen. *Cancer Res.*, **52**, 1360-1363.

McCLAY, E.F., MASTRANGELO, M.J., BELLET, R.E. & BERD, D. (1987). Combination chemo/hormonal therapy in the treatment of malignant melanoma. *Cancer Treat. Rep.*, **71**, 465-469.

McCLAY, E.F., MASTRANGELO, M.J., SPRANDIO, J.D., BELLET, R.E. & BERD, D. (1989). The importance of tamoxifen to a cisplatin containing regimen in the treatment of metastatic melanoma. *Cancer*, **63**, 1293-1295.

McCLAY, E.F., MASTRANGELO, M.J., BERD, D. & BELLET, R.E. (1992*a*). Effective combination chemo/hormonal therapy for malignant melanoma: experience with three consecutive trials. *Int. J. Cancer*, **50**, 553-556.

McCLAY, E.F., CHRISTEN, R., ALBRIGHT, K.A., JONES, J.A., EASTMAN, A. & HOWELL, S.B. (1992*b*). Modulation of cisplatin resistance in human malignant melanoma cells. *Cancer Res.*, **52**, 6790-6796.

McCLAY, E.F., McCLAY, M.E., ALBRIGHT, K.A., JONES, J.A., CHRISTEN, R., ALCARAZ, J. & HOWELL, S.B. (1993*a*). Tamoxifen modulation of cisplatin resistance in patients with metastatic melanoma. *Cancer*, **72**, 1914-1918.

McCLAY, E.F., ALBRIGHT, K.A., JONES, J.A., CHRISTEN, R. & HOWELL, S.B. (1993*b*). Tamoxifen modulation of cisplatin sensitivity in human malignant melanoma cells. *Cancer Res.*, **53**, 1571-1576.

McCLAY, E.F., ALBRIGHT, K.D., JONES, J.A., CHRISTEN, R.D. & HOWELL, S.B. (1993*c*). Tamoxifen modulation of cisplatin cytotoxicity in human malignancies. *Int. J. Cancer*, **55**, 1012-1022.

McCLAY, E.F., ALBRIGHT, K.D., JONES, J.A., CHRISTEN, R.D. & HOWELL, S.B. (1993*d*). N,N-diethyl-2-[(4-phenylmethy)-phenoxyl]-ethanamine HCL (DPPE) is synergistic with cisplatin (DDP) in human melanoma cell lines. *Proc. Am. Assoc. Cancer Res.*, **34**, 402.

TAETLE, R., JONES, O., HONEYSETT, J., ABRAMSON, I., BRADSHAW, C. & REID, S. (1987). Characterization of xenograft-derived melanoma cell lines. *Cancer*, **60**, 1836-1841.